# Laparoscopic median arcuate ligament release: Successful management of Dunbar syndrome in a young male

**DOI:** 10.1016/j.ijscr.2025.112109

**Published:** 2025-10-26

**Authors:** Sujan Paudel, Prajjwol Luitel, Abhishek Kumar Shah, Bikal Ghimire

**Affiliations:** a Maharajgunj Medical Campus, Institute of Medicine, Tribhuvan University Teaching Hospital, Kathmandu, Nepal; b Department of General Surgery, Maharajgunj Medical Campus, Institute of Medicine, Tribhuvan University Teaching Hospital, Kathmandu, Nepal

**Keywords:** Dunbar syndrome, Celiac artery compression, Laparoscopic release, Postprandial pain, Resource-limited settings

## Abstract

**Introduction and importance:**

Median arcuate ligament syndrome (MALS), also known as Dunbar syndrome, is a rare condition caused by compression of the celiac artery and/or ganglion by the median arcuate ligament. Its incidence is poorly defined, but it typically affects lean, middle-aged women. Due to its non-specific symptoms, MALS is often underdiagnosed. Although open surgery is conventional in resource-limited settings, laparoscopic approaches offer favorable outcomes.

**Presentation of case:**

We report a case of a 24-year-old male with chronic postprandial upper abdominal pain and significant weight loss. Imaging via contrast-enhanced CT confirmed celiac artery compression consistent with MALS. He underwent successful laparoscopic median arcuate ligament release with celiac neurolysis.

**Discussion:**

MALS presents diagnostic challenges due to its overlapping symptoms with other gastrointestinal disorders. Imaging and clinical correlation are crucial for diagnosis. While open surgery remains common in low-resource settings, laparoscopy provides reduced morbidity and quicker recovery.

**Conclusion:**

This case highlights the importance of considering MALS in chronic postprandial pain and supports the feasibility of laparoscopic decompression as an effective treatment, even in resource-constrained environments.

## Highlights

• 24-year-old male with postprandial pain, weight loss, diagnosed as Median Arcuate Ligament Syndrome.
• Contrast CT confirmed celiac artery compression consistent with MALS.
• Laparoscopic ligament release with celiac neurolysis done successfully.
• Minimally invasive surgery proved feasible even in resource-limited setup.
• Uneventful recovery; patient symptom-free at 6-month follow-up.

## 1 Introduction

Median arcuate ligament syndrome (MALS), also known as Dunbar syndrome or celiac axis compression syndrome, results from compression of the celiac axis and/or celiac ganglion by the median arcuate ligament of the diaphragm [[1], [2], [3], [4]]. The incidence is estimated at 2 per 100,000, most commonly affecting thin women aged 40–60 years [5,6]. It classically presents with a triad of postprandial pain, weight loss and bruit in the epigastrium [7]. Non-specific and variable symptoms contribute to frequent underdiagnosis. Traditionally, open surgery is preferred, especially in resource-limited settings [8], but this case demonstrates the successful use of a laparoscopic approach for the treatment of Dunbar syndrome. This case report is done in accordance with SCARE guidelines [9].

## 2 Case presentation

A 24-year-old male was referred to our center with a 2-year history of chronic upper abdominal pain and discomfort, predominantly occurring after meals. He also experienced a marked decrease in food intake, resulting in an 8 kg weight loss over the past year. The patient had no other significant medical history or comorbidities. He used over-the-counter antacids with only partial relief.

On physical examination, the patient had a body mass index (BMI) of 18 kg/m^2^, indicating underweight status. Systemic examination was unremarkable.

Laboratory investigations revealed the following: a total leukocyte count of 7600/mm^3^ (normal range: 4000–11,000/mm^3^), hemoglobin of 14.5 g/dL (normal range: males 13.5–17.5 g/dL), and a platelet count of 165,000/mm^3^ (normal range: 150,000-400,000/mm^3^). Renal function tests showed blood urea nitrogen of 26.6 mg/dL (normal range: 7–20 mg/dL) and creatinine of 1.0 mg/dL (normal range: 0.7–1.3 mg/dL). Serum sodium was 136 mmol/L (normal range: 136–145 mmol/L) and potassium was 4.0 mmol/L (normal range: 3.5–5.1 mmol/L). Coagulation studies were within normal limits, with a prothrombin time of 14 s (normal range: 11–13.5 s) and an international normalized ratio (INR) of 1.0 (normal range: 0.9–1.2). Upper gastrointestinal endoscopy and an ultrasound of the abdomen and pelvis were unremarkable.

**Fig. 1:**
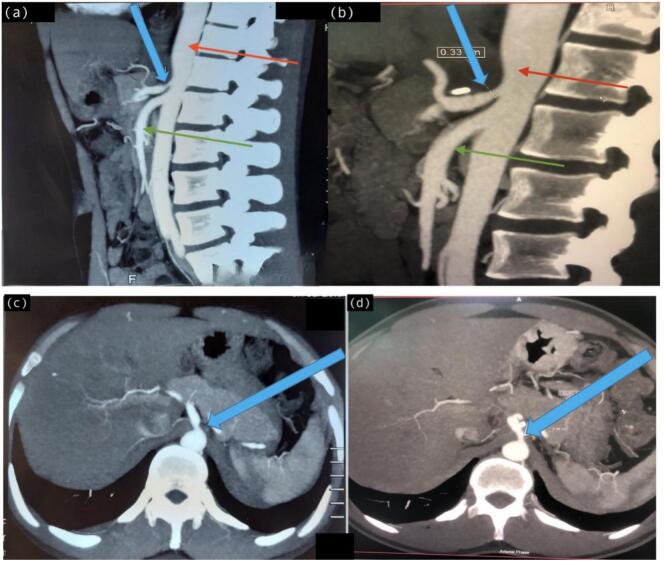
(a-d): Pre-operative (a, c) and post-operative (b, d) CECT images of a patient with Dunbar syndrome. (a) Pre-operative sagittal image shows compression of the celiac artery (blue arrow) by the median arcuate ligament, with the abdominal aorta (red arrow) and superior mesenteric artery (green arrow) visible. (b) Post-operative sagittal image reveals increased luminal diameter of the celiac artery (blue arrow) after laparoscopic release, with a diameter of 0.33 cm. (c) Pre-operative axial image highlights the celiac artery compression (blue arrow). (d) Post-operative axial image shows improved caliber of the celiac artery (blue arrow), confirming successful decompression following surgery. (For interpretation of the references to colour in this figure legend, the reader is referred to the web version of this article.)

Subsequent imaging via contrast-enhanced computed tomography (CECT) of the abdomen and pelvis demonstrated radiographic evidence of celiac artery compression, consistent with the diagnosis of Dunbar syndrome. Preoperative sagittal CECT images demonstrated significant narrowing of the proximal celiac artery with a characteristic hooked appearance and post-stenotic dilatation, suggestive of median arcuate ligament compression (Fig. 1a). The preoperative luminal diameter at the most stenotic point was approximately 0.18 cm (Fig. 1c). Other potential causes of celiac artery stenosis such as atherosclerosis, arteritis, and external compressive masses were excluded based on clinical history, normal laboratory work-up, and absence of arterial wall irregularities or calcification on CT imaging.

**Fig. 2:**
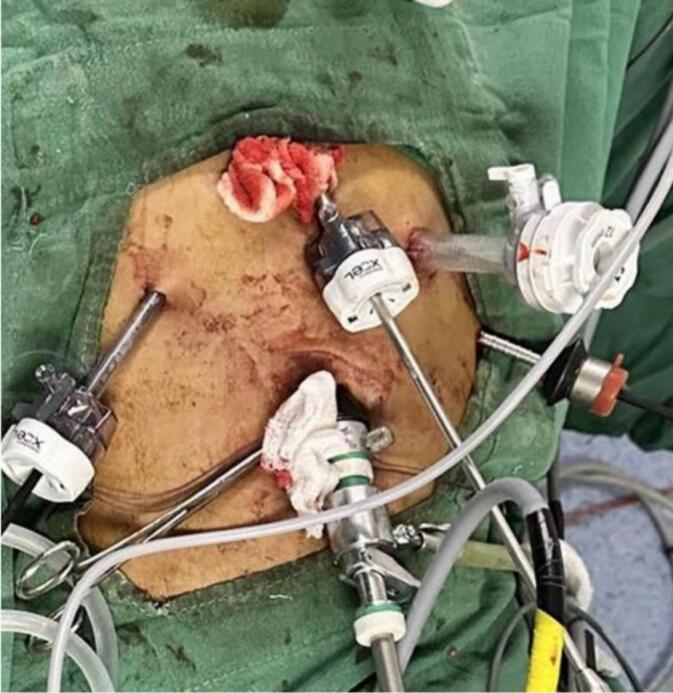
Illustrates the port placement for laparoscopic median arcuate ligament decompression. A 10 mm umbilical port is positioned for the camera, with an epigastric port located above it for dissection. Two 5 mm lateral ports in the upper quadrants provide access for retraction and additional instruments, while a subxiphoid port offers enhanced access for the procedure.

The patient underwent laparoscopic median arcuate ligament release under general anesthesia. He was positioned supine on a split-leg operating table, with the operator standing between the legs and one assistant on either side. The laparoscopic procedure involved the placement of ports as shown in (Fig. 2).

**Fig. 3:**
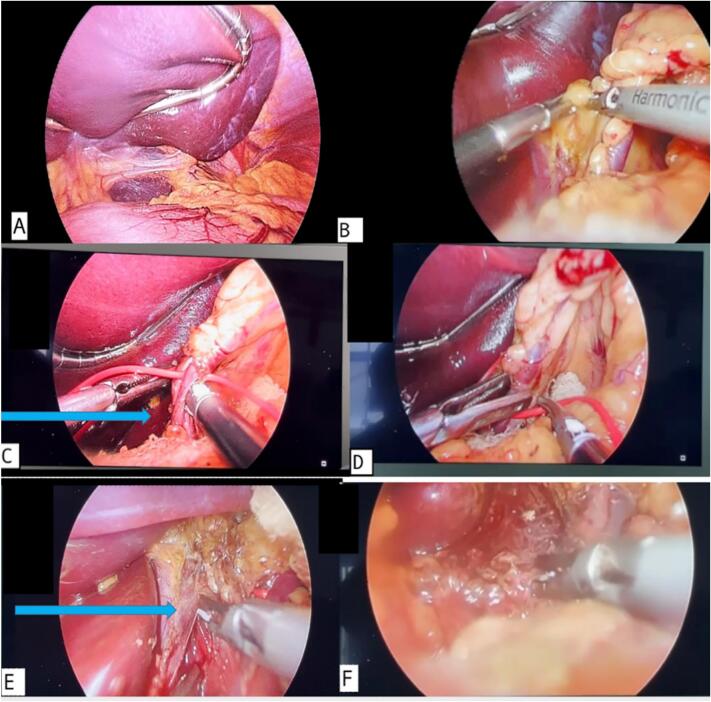
Laparoscopic steps in the surgical treatment of Dunbar syndrome. (A) The left lobe of the liver is elevated to provide adequate exposure of the operative field. (B) Incision of the gastrohepatic ligament is performed to access the celiac axis. (C) The left gastric artery is exposed up to its root (indicated by the blue arrow) to visualize the celiac trunk. (D) The dissection is carried cephalad, allowing further exposure of the celiac artery and its branches. (E) The median arcuate ligament is divided (blue arrow indicates the site of division), relieving compression on the celiac artery. (F) Celiac neurolysis is performed to ensure complete decompression of the celiac axis, with visible neurovascular structures following the release. (For interpretation of the references to colour in this figure legend, the reader is referred to the web version of this article.)

It was followed by elevation of the left liver lobe using a liver retractor. The gastro-hepatic ligament was incised, and dissection proceeded in a caudal-to-cephalad direction to expose the left gastric vessels and the celiac trunk. Further dissection exposed the left gastric artery and vein, avoiding the hiatus, and the muscle fibers overlying the aorta were gradually exposed to create a surgical window while avoiding injury to the aorta. The median arcuate ligament was then divided, and celiac neurolysis was performed. Celiac neurolysis was performed by carefully dissecting and cauterizing the surrounding fibrous and neural tissue encasing the celiac axis, thereby reducing neural compression and contributing to symptomatic relief [Fig. 3(a-f)].

Postoperative CECT imaging performed after the laparoscopic release revealed a significant increase in the celiac artery's luminal diameter. The diameter improved from 0.18 cm preoperatively to 0.33 cm postoperatively (Fig. 1b), confirming successful surgical decompression. The postoperative axial images also demonstrated improved caliber of the celiac artery, indicating effective surgical intervention (Fig. 1d).

**Fig. 4:**
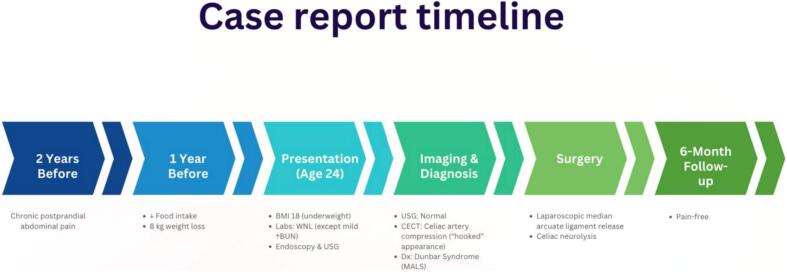
Timeline of clinical course showing symptom onset, diagnostic evaluation, laparoscopic treatment, and 6-month follow-up in a 24-year-old male with MALS.

The patient had an uneventful postoperative course and was discharged on the second postoperative day. Complete resolution of his preoperative pain was noted following the surgery. On follow-up at 6 months, the patient did not have abdominal complaints. A summary of the patient's clinical course, including symptoms, diagnostic workup, surgical intervention, and follow-up, is presented in the timeline (Fig. 4).

## 3 Discussion

Dunbar syndrome is a rare constellation of clinical signs and symptoms associated with celiac artery compression by the median arcuate ligament (MAL). The pathophysiology of Dunbar syndrome remains controversial [8,10]. Two main etiological theories have been proposed to explain the symptoms, which include a vascular and a neurogenic cause, respectively. According to the vascular theory, mesenteric vascular compression produces mesenteric ischemia, creating abdominal angina and other related symptoms. In contrast, the neurogenic theory posits that the splanchnic vasoconstriction is due to the stimulation of the celiac ganglion and the celiac plexus [11].

The true prevalence of MALS is unclear, which is partly due to its variable clinical presentation. There is a tendency toward the female phenotype (4:1), and the median age typically lies between 30 and 50 years [12]. Cases have also been reported in pediatric populations [13]. Frequently reported symptoms include epigastric pain, nausea, vomiting, weight loss, and postprandial or exercise-induced abdominal pain. Clinical signs may include abdominal bruits (amplified with expiration), epigastric tenderness, and marked weight loss. Due to the non-specific nature of symptoms, MALS is often misdiagnosed or attributed to more common gastrointestinal disorders such as gastritis, gastroenteritis, kidney stones etc. [14]. Our patient was initially treated for other causes of postprandial pain like peptic ulcer disease before imaging studies confirmed the diagnosis of Dunbar syndrome. The delay in diagnosis is consistent with existing literature, where patients often undergo extensive evaluations before MALS is considered [14].

Diagnosis of MALS typically depends on exclusion of alternative causes of abdominal pain. Indeed, before investigation for MALS, a formal gastroenterology review is advisable to rule out more common explanations for the patients' symptoms; esophagogastroduodenoscopy, colonoscopy, motility studies, and cross-sectional imaging and relevant hematologic studies should be obtained [15]. When clinical suspicion of chronic mesenteric ischemia arises, duplex ultrasound (DUS) is the first-line recommended investigation [8]. According to the European Society for Vascular Surgery (ESVS) clinical practice guidelines (2025), duplex ultrasound and CTA are complementary first-line modalities to map occlusive disease and to exclude other intra-abdominal causes; intervention is recommended only for symptomatic patients after alternative etiologies have been excluded [8]. But in our case, diagnosis was made through axial and sagittal contrast-enhanced CT (CECT) which showed compression of the celiac artery (blue arrow) by the median arcuate ligament, with the abdominal aorta (red arrow) and superior mesenteric artery (green arrow) visible, guided by clinical suspicion Fig. 1(b) and (d). Further complicating the diagnosis, the severity of symptoms does not always correlate with the degree of vascular compression seen on imaging [15,16]. Compression of the celiac artery can be a normal anatomical variant in asymptomatic individuals, necessitating careful correlation with clinical findings to avoid misdiagnosis [16].

The most recent 2025 European Society for Vascular Surgery (ESVS) clinical practice guidelines further reinforce a multimodal, symptom-driven approach to suspected celiac axis compression [17]. The ESVS advises that imaging findings alone should not determine management and recommends combining careful clinical assessment with targeted noninvasive imaging (duplex ultrasound and CTA) to exclude atherosclerotic, inflammatory or extrinsic causes before intervention. For patients with mild or equivocal symptoms, conservative management and close clinical surveillance are favoured, whereas invasive treatment is reserved for patients with persistent, functionally limiting symptoms after alternative diagnoses have been excluded. These recommendations support our strategy of correlating the characteristic sagittal CTA appearance with the patient's clinical syndrome before proceeding to laparoscopic decompression, and of planning follow-up imaging only when clinically indicated.

Celiac artery stenosis seen on imaging is not pathognomonic for median arcuate ligament syndrome (MALS) — in fact, anatomical compression of the celiac trunk by the median arcuate ligament is observed in 10–24 % of asymptomatic individuals [18]. Several other entities may cause or mimic celiac trunk narrowing, including atherosclerosis, large-vessel vasculitides (e.g., Takayasu arteritis), fibromuscular dysplasia, or extrinsic compression from pancreatic masses, lymph nodes, or retroperitoneal fibrosis [17]. Because of this, MALS is considered a diagnosis of exclusion [19]. In our patient, we systematically excluded alternative explanations: laboratory evaluation showed no markers of systemic inflammation or autoimmune disease; upper GI endoscopy and abdominal ultrasonography revealed no evidence of peptic ulcer, biliary or pancreatic disease; and imaging revealed no arterial wall calcification, plaque, or mural irregularity to suggest atherosclerosis. The clinical picture (postprandial pain, weight loss) and the characteristic “hooked” contour of the celiac trunk on sagittal CT supported MALS. Importantly, the marked symptomatic relief after surgical decompression further corroborates that this was not an incidental finding but a clinically significant MALS.

Generally, treatment of Dunbar syndrome involves releasing the compression of the celiac axis by sectioning of the median arcuate ligament [20,21]. It may be performed via open, laparoscopy, or robotic-associated laparoscopy [22,23]. Robotic-associated laparoscopy is not possible in a resource-limited setting due to cost limitations. Although laparoscopic treatment has become the standard treatment in most settings, open surgery is still preferred in resource-limited settings. In our resource-limited setting, the decision for a laparoscopic approach was guided by a cost–benefit analysis. While the initial setup cost for laparoscopic equipment is significant, the long-term benefits for both the patient and the healthcare system are substantial [24]. Compared to open surgery, which requires a large upper abdominal incision, laparoscopy offers reduced postoperative pain, lower analgesic requirements, a shorter hospital stay (2 days in our case versus an estimated 4.6 ± 1.7 days for open surgery), and a quicker return to daily activities and work [25]. These factors mitigate the indirect costs of lost productivity and reduce the burden on hospital resources, such as nursing care and inpatient beds. Although open ligament release remains a viable and more familiar option in many low-resource environments, the laparoscopic approach, once expertise is gained, provides superior patient outcomes with lower overall hospital cost when considering the entire episode of care [26].

In some cases, persistent stenosis of the celiac artery after decompression may necessitate further intervention, such as arterial reconstruction (aortoceliac bypass or a celiac artery patch angioplasty) or percutaneous transluminal angioplasty with or without stenting [15]. However, in this patient, adequate sectioning of the MAL was confirmed by the increased luminal diameter of the celiac artery on post-operative computed tomography (CT) images, and the patient's symptoms were fully alleviated following laparoscopic release, thus avoiding the need for arterial reconstruction. Along with surgical decompression of MAL, celiac ganglionectomy, rather than simple neurolysis, is recommended to address the neuropathic pain often associated with MALS [15]. But in this case, simple neurolysis was performed successfully since celiac ganglionectomy requires advanced surgical skills and equipment, which was not available in our setting.

Regarding long-term follow-up, the patient in this report remained symptom-free at the 6-month mark. However, we acknowledge that sustained follow-up is crucial for MALS patients. Reported rates of sustained symptomatic improvement range widely (~85–90 % in contemporary series) while estimates of recurrence (4–6.5 % recurrence rate) depending on selection criteria and follow-up duration [27,28]. Accordingly, structured follow-up is recommended. Routine imaging is not necessary in asymptomatic individuals; however, noninvasive modalities such as duplex ultrasonography or CTA should be performed if symptoms recur to evaluate for residual or recurrent stenosis [17]. In our case, we advised annual clinical evaluation for at least 3 years, reserving imaging for patients who develop recurrent symptoms, in line with current ESVS recommendations [17].

## 4 Conclusion

Median arcuate ligament syndrome (MALS) is a rare and diagnostically challenging condition, as celiac artery compression may be incidental and its symptoms often mimic more common gastrointestinal or vascular disorders. Accurate diagnosis requires close clinico-radiological correlation and systematic exclusion of other causes. It demonstrates that laparoscopic decompression, though resource-intensive, is feasible in low-resource settings and offers faster recovery, shorter hospital stay, and reduced indirect costs compared with open surgery. Adjunctive neurolysis can further enhance symptom relief when formal ganglionectomy is not possible, as shown in this patient.

## Author contribution

Contribution of each author

Sujan Paudel: study concept, writing the paper, revision

Prajjwol Luitel: study concept, writing the paper, revision

Abhishek Shah: writing the paper, revision

Bikal ghimire: study concept, visualization and supervision, patient management

## Consent

Written Informed consent was obtained from the patient for the publication of this case report, including the use of de-identified imaging and clinical data. A copy of the written consent is available for review by the Editor-in-chief of this journal on request.

## Ethical approval

Since this is a case report, our Institutional Review Board Institute of Medicine (IOM) has waived the requirement for ethical approval.

## Guarantor

Bikal Ghimire.

## Research registration number

None.

## Provenance and peer review

The paper is not commissioned, externally peer-reviewed.

## Funding

No funding received.

## Conflict of interest statement

The authors declare that they have no competing interests.

## References

[1] Balzan S.M.P., Gava V.G., Pedrotti S., Magalhães M.A., Schwengber A., Dotto M.L. (Oct 21 2019). Prevalence of hepatic arterial variations with implications in pancreatoduodenectomy. Arq. Bras. Cir. Dig..

[2] De’Ath H.D., Wong S., Szentpali K., Somers S., Peck T., Wakefield C.H. (Nov 2018). The laparoscopic management of median arcuate ligament syndrome and its long-term outcomes. J. Laparoendosc. Adv. Surg. Tech. A.

[3] Rebelos E., Cipriano A., Ferrini L., Trifirò S., Napoli N., Santini M. (Jul 1 2019). Spontaneous bleeding of the inferior pancreatic-duodenal artery in median arcuate ligament syndrome: do not miss the diagnosis. Oxf. Med. Case Rep..

[4] Santos P.V.D., Barbosa A.B.M., Targino V.A., Silva N. de A., Silva Y.C. de M., Barbosa F. (Dec 6 2018). Anatomical variations of the celiac trunk: a systematic review. Arq. Bras. Cir. Dig..

[5] Grotemeyer D., Duran M., Iskandar F., Blondin D., Nguyen K., Sandmann W. (Nov 2009). Median arcuate ligament syndrome: vascular surgical therapy and follow-up of 18 patients. Langenbecks Arch. Surg..

[6] Kulich K.R., Malfertheiner P., Madisch A., Labenz J., Bayerdörffer E., Miehlke S. (Oct 28 2003). Psychometric validation of the German translation of the Gastrointestinal Symptom Rating Scale (GSRS) and Quality of Life in Reflux and Dyspepsia (QOLRAD) questionnaire in patients with reflux disease. Health Qual. Life Outcomes.

[7] Harjola P.T. (1963). A rare obstruction of the coeliac artery. Report of a case. Ann. Chir. Gynaecol. Fenn..

[8] Björck M., Koelemay M., Acosta S., Bastos Goncalves F., Kölbel T., Kolkman J.J. (Apr 2017). Editor’s choice - management of the diseases of mesenteric arteries and veins: clinical practice guidelines of the European Society of Vascular Surgery (ESVS). Eur. J. Vasc. Endovasc. Surg..

[9] Kerwan A., Al-Jabir A., Mathew G., Sohrabi C., Rashid R., Harvard T.H. Chan School of Public Health, Boston, USA (2025). Revised Surgical Case Report (SCARE) guideline: an update for the age of Artificial Intelligence. Prem. J. Sci..

[10] Gander S., Mulder D.J., Jones S., Ricketts J.D., Soboleski D.A., Justinich C.J. (Feb 2010). Recurrent abdominal pain and weight loss in an adolescent: celiac artery compression syndrome. Can. J. Gastroenterol..

[11] Bobadilla J.L. (Aug 2013). Mesenteric ischemia. Surg. Clin. North Am..

[12] Kim E.N., Lamb K., Relles D., Moudgill N., DiMuzio P.J., Eisenberg J.A. (May 1 2016). Median arcuate ligament syndrome-review of this rare disease. JAMA Surg..

[13] Scholbach T. (Mar 2006). Celiac artery compression syndrome in children, adolescents, and young adults: clinical and color duplex sonographic features in a series of 59 cases. J. Ultrasound Med..

[14] Abu-Hilal A.H.H., Adawi Y., Abu-Ghosh M., Abu-Hilal L.H., Al Shawwa K.N., AbuKeshek T. (Jan-Dec 2023). A case series of median arcuate ligament syndrome with varied presentations. J. Investig. Med. High Impact Case Rep..

[15] Goodall R., Langridge B., Onida S., Ellis M., Lane T., Davies A.H. (Jun 2020). Median arcuate ligament syndrome. J. Vasc. Surg..

[16] Baskan O., Kaya E., Gungoren F.Z., Erol C. (Aug 2015). Compression of the celiac artery by the median arcuate ligament: multidetector computed tomography findings and characteristics. Can. Assoc. Radiol. J..

[17] Koelemay M.J., Geelkerken R.H., Kärkkäinen J., Leone N., Antoniou G.A., de Bruin J.L. (Aug 2025). Editor’s choice - European Society for Vascular Surgery (ESVS) 2025 Clinical Practice Guidelines on the management of diseases of the mesenteric and renal arteries and veins. Eur. J. Vasc. Endovasc. Surg..

[18] Koç M., Artaş H., Serhatlıoğlu S. (Dec 12 2018). The investigation of incidence and multidetector computed tomography findings of median arcuate ligament syndrome. Turk. J. Med. Sci..

[19] Saleem T., Katta S., Baril D.T. (2023). StatPearls [Internet].

[20] Fajer S., Cornateanu R., Ghinea R., Inbar R., Avital S. (Dec 2014). Laparoscopic repair of median arcuate ligament syndrome: a new approach. J. Am. Coll. Surg..

[21] Ramakrishnan P., Deuri B., Keerthi M.S.S., Naidu S.B., Subbaiah R., Raj P. (Apr 2016). Laparoscopic division of median arcuate ligament for the celiac axis compression syndrome-two case reports with review of literature. Indian J. Surg..

[22] Coelho J.C.U., da Silva J.C., Domingos M.F., Paulin J.A.N., Ferronato G.F. (Nov-Dec 2015). Laparoscopic treatment of celiac axis compression syndrome: case report. Arq. Bras. Cir. Dig..

[23] Roberts B., Pevsner R., Alkhoury F. (Jan 2020). Robotic approach for median arcuate ligament release in pediatrics. J. Laparoendosc. Adv. Surg. Tech. A.

[24] Dorricott A.R., Dickinson A., McNickle A.G., Batra K., Flores C.E., Fraser D.R. (Sep 2024). Trauma laparoscopy: time efficient, cost effective, and safe. J. Surg. Res..

[25] Alsabbagh Y., Lanka S.P., Jlilati A., Sarmiento J., Jacobs C., Elli F. (Jun 2025). Open versus laparoscopic median arcuate ligament release: a single-institution experience. J. Vasc. Surg..

[26] Alnahhal K.I., Tedesco A., Khan Z.Z., Irshad A., Salehi P. (Oct 2023). Median arcuate ligament syndrome: comparing the safety of open and laparoscopic management in a large cohort. Ann. Vasc. Surg..

[27] Jimenez J.C., Harlander-Locke M., Dutson E.P. (Sep 2012). Open and laparoscopic treatment of median arcuate ligament syndrome. J. Vasc. Surg..

[28] Kazmi S.S.H., Safi N., Berge S.T., Kazmi M., Sundhagen J.O., Hisdal J. (Mar 24 2022). Laparoscopic surgery for median arcuate ligament syndrome (MALS): a prospective cohort of 52 patients. Vasc. Health Risk Manag..

